# Supplementary material for: Mutations in PCYT2 disrupt etherlipid biosynthesis and cause a complex hereditary spastic paraplegia
Source: Brain. 2019 Oct 22;142(11):3382–97. doi: 10.1093/brain/awz291 (PMC6821184; doi:10.1093/brain/awz291)
Supplement: awz291_Supplementary_Data [file awz291_supplementary_data.zip › awz291-Suppl_data/Supplementary_Data4.zip!/Supplementary information 4 - plasma lipidomics.zip!/Plasma lipidomics/Control vs Patient/Statistical plots and data/OPLS-DA.pdf]

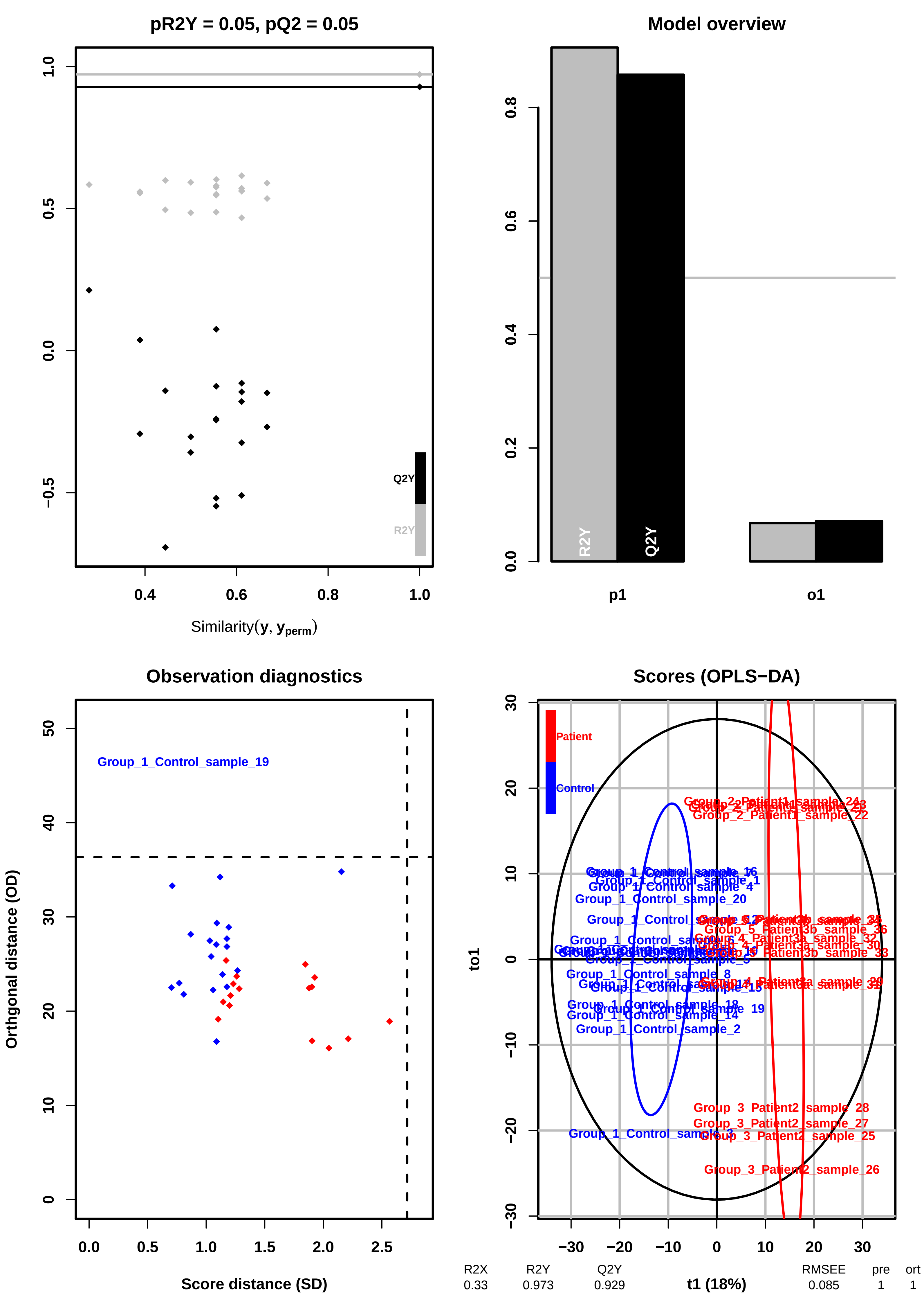

pR2Y = 0.05, pQ2 = 0.05
Q2Y
R2Y
Similarity(y, yperm)
Model overview
R2Y
Q2Y
p1
o1
Observation diagnostics
Group_1_Control_sample_19
Orthgonal distance (OD)
Score distance (SD)
Scores (OPLS−DA)
Patient
Control
Group_2_Patient1_sample_22
Group_1_Control_sample_4
Group_1_Control_sample_20
Group_5_Patient3b_sample_36
Group_4_Patient3a_sample_32
Group_4_Patient3a_sample_30
Group_5_Patient3b_sample_33
Group_1_Control_sample_5
Group_1_Control_sample_8
Group_1_Control_sample_15
Group_1_Control_sample_18
Group_1_Control_sample_14
Group_1_Control_sample_2
Group_3_Patient2_sample_28
Group_3_Patient2_sample_27
Group_3_Patient2_sample_25
Group_3_Patient2_sample_26
to1
t1 (18%)
R2X 0.33
R2Y 0.973
Q2Y 0.929
RMSEE 0.085
pre 1
ort 1
